# Physical and chemical characterisation of ophthalmic lens-grinding wastewater: uncovering environmental implications

**DOI:** 10.1007/s11356-026-37708-w

**Published:** 2026-04-01

**Authors:** Juliana Araújo, Pedro Ramos, Elsa Silvestre, Artur Mateus, Anabela Massano, Timur Nikitin, Rui Fausto, Pedro S. P. Silva, António J. Guiomar, Pedro F. Cruz, Abílio J. F. N. Sobral, Telma Encarnação

**Affiliations:** 1 Department of Chemistry, CQC-IMS, University of Coimbra, 3004-535 Coimbra, Portugal; 2 PTScience, Avenida Do Atlântico, N 16, Office 5.07, Parque das Nações, 1990-019 Lisboa Portugal; 3 Institute for Sustainability and Innovation in Structural Engineering, Department of Civil Engineering, University of Coimbra, 3030-788 Coimbra, Portugal; 4 Opticentro, 2460-071 Alcobaça, Portugal; 5 Centre for Rapid and Sustainable Product Development, Polytechnic Institute of Leiria, 2430-028 Marinha Grande, Portugal; 6 Faculty Sciences and Letters, Department of Physics, Istanbul Kultur University, 34158 Bakirkoy, Istanbul, Turkey; 7 Department of Physics, CFisUC, University of Coimbra, 3004-516 Coimbra, Portugal; 8 Department of Life Sciences, CERES, University of Coimbra, 3000-456 Coimbra, Portugal

**Keywords:** Ophthalmic industry, Optical stores, Grinding process, Waste lenses, Ophthalmic lenses, Endocrine-disrupting chemicals, Emerging pollutants, Nanoplastics

## Abstract

The ophthalmic industry’s eyewear lens production generates substantial waste during the lens grinding processes, contributing to significant environmental challenges. This process consumes approximately 20 L of water per eyeglass lens pair, which is subsequently discharged directly into wastewater systems, releasing a complex mixture of pollutants, including micro- and nanoplastics, into the environment. This study investigates the wastewater generated by lens grinding machines within the optical retail sector and provides a detailed physical and chemical characterisation of this waste stream. The results revealed a significant material loss during grinding, with approximately 50% of each initial lens mass being discharged during cutting. Based on conservative estimates, 5,770 tonnes of plastic waste are released into the environment each year, the equivalent of filling and discarding a marine container every 42 h. RAMAN analysis identified TiO_2_ in both anatase and rutile crystalline forms in the lens coatings, both of which are recognised as emerging environmental pollutants. The characteristic N–H stretching vibrations of polyurethanes were detected in the FTIR analysis. Other hazardous contaminants, including particles below 2 µm, heavy metals, bisphenol A, phthalates, and fluorine-containing compounds, were also identified through SEM, NMR, and GC-MS. The presence of these substances raises significant concerns, as they are known endocrine-disrupting chemicals and persistent organic pollutants, which pose a significant risk to aquatic ecosystems and human health. Our findings revealed that while global lens consumption is driven by diverse regional preferences and multiple factors, waste generation is inherently systemic and independent of consumer choice, representing a structural technological constraint of current optical manufacturing processes. This study provides new, valuable information to alert the practitioners, competent authorities, policy makers, and society, supporting the development of future policies, alternative materials, and improved waste management strategies within the downstream ophthalmic value chain.

**Supplementary Information:**

The online version contains supplementary material available at 10.1007/s11356-026-37708-w.

## Introduction

The number of people with vision disorders is rapidly increasing due to factors such as an ageing population and increased exposure to digital devices, contributing to a rise in the purchase and production of ophthalmic eyewear (Boyd 2024; Chu et al. 2023; Holden et al. 2016; Kaur et al. 2022). This is evidenced by the significant growth reported by major eyewear industry companies, with year-on-year sales increases exceeding 13.9% (“FY 2022 Results. EssilorLuxottica”). The global eyewear market, valued at approximately $181.75 billion in 2025, is projected to expand at a compound annual growth rate (CAGR) of 7% from 2026 to 2034, reaching $330.08 billion (Insights); this emphasise the influence of these markets on society (*Spectacle Lens Market Size & Share Analysis—Growth Trends & Forecasts*). This increase in lens production implies an increased environmental burden; the processing of lenses involves surfacing and edging the material, resulting in the generation of significant quantities of nano- and microplastics, as well as inorganic and organic pollutants. As the market grows, these pollutants will increase proportionally, leading to long-term ecological challenges. This reveals the urgent need for sustainable solutions in the management of this by-product waste. Current lens grinding entails the shaping, cutting, and polishing of lenses to achieve the desired curvatures, thicknesses, and contours. This ensures that the lenses fit the frames and meet the required prescription or functional specifications. This process generates substantial waste, as post-cutting lens material is typically discarded rather than reused or recycled. Furthermore, these processes consume significant water, with an estimated 4–5 L per minute during grinding, often excluded from closed-loop water recycling systems (Essilor Instruments USA. Essilor KAPPA CTD L07 M15—Instructions Manual). This trend is also evident in optical store lens manufacturing, where lenses are shaped for spectacles via grinding. Studies indicate approximately 20 L of water are used to bevel a single pair of eyeglass lenses (J. Lee et al. 2021). Critically, untreated wastewater is often discharged directly into municipal wastewater systems, releasing nanoplastics (NPs), microplastics (MPs), and chemical constituents. Previous research has quantified NP concentrations in lens-edging wastewater at 0.0136–0.0324 mg per litre, translating to an estimated 4.08 mg of NPs per day per optical store. This cumulative effect leads to the release of approximately 57 g of NPs daily, based on an estimated 14,000 operational optical stores at the time of the study (J. Lee et al. 2021) Furthermore, these MPs can release high levels of inorganic and organic contaminants into water (Novotna et al. 2023).

**Fig. 1:**
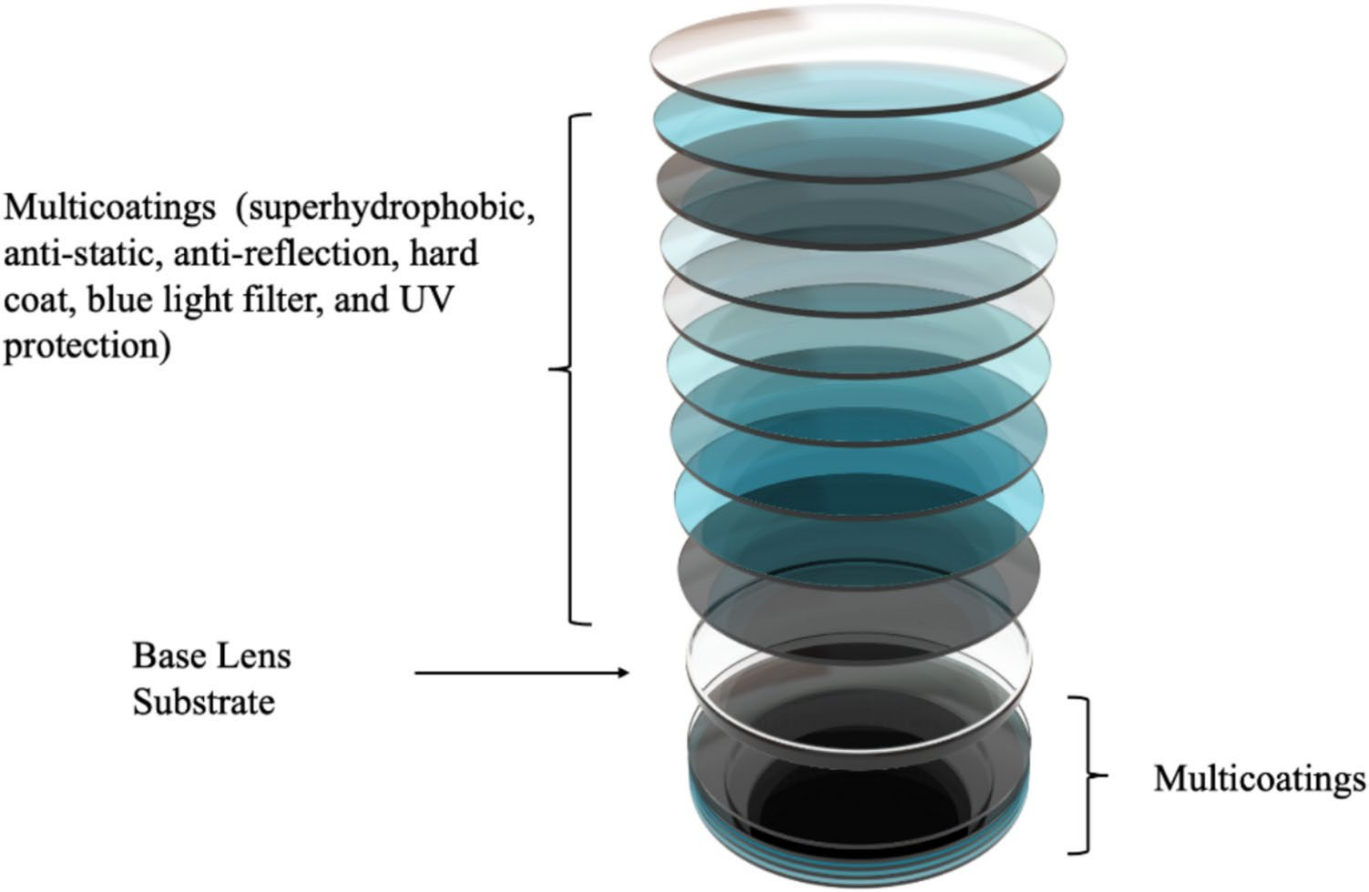
Schematic illustration of multicoated ophthalmic lens

Beyond its polymeric matrix (comprising acrylics, polythiourethanes, polycarbonates, polystyrenes, or polysulfones), ophthalmic lenses incorporate diverse additives and coatings (see Fig. 1). These include endocrine-disrupting chemicals, which can interfere with human and animal endocrine systems and degrade aquatic ecosystems and wildlife, including bisphenol A, benzene, toluene, and heavy metals, (J. Lee et al. 2021) as well as mould release agents, UV absorbers, tints, photochromic dyes, optical brighteners, plasticisers, light and thermal stabilisers, and oxidants (Dyball 1980; Okoroafor et al. 2001; Richard, Primel, & Yean, 2008).

Heavy metals, including cadmium, chromium, and lead, are prevalent environmental pollutants. Their environmental persistence leads to bioaccumulation within the food chain, posing significant health risks (Balali-Mood et al. 2021).

Ophthalmic waste presents substantial health and environmental challenges, including risks to the health of living organisms, the introduction of harmful chemicals into the food chain, and the wasteful consumption of large amounts of water that could otherwise be treated and repurposed (Encarnação et al. 2020; J. Lee et al. 2021).

Even though the knowledge on the occurrence and properties of microplastics and nanoplastics (MNPLs) is rapidly increasing, their sources remain largely unexplored (Roslan et al. 2024). One such source that is particularly under-researched is grinding wastewater. Despite the growing environmental risks associated with this wastewater, such as high-water consumption, microplastic pollution, and endocrine-disrupting chemicals, a comprehensive characterisation of the grinding matrix is still lacking. The study aims to bridge this gap by quantifying the waste released of lens grinding and defining its chemical and particle profile. By identifying these specific pollutants, this research provides the robust data necessary to inform sustainable waste management policies and the development of eco-friendly alternatives.

## Materials and methods

### Lens edging

To analyse the waste generated during the lens grinding process, 187 spectacle lenses of 12 different commercial types (see Table 1) were processed using an Essilor Kappa CTD M15 Edger (Essilor International S.A., Paris, France) in Opticentro, Alcobaça-Portugal. The waste sample was derived from real sales and real customers with various visual impairments and was collected over a period of one month, reflecting the real-world variability of lens materials and prescriptions encountered in a commercial optical store.

**Table 1:** Diverse lens types used physical and chemical characterisation

Lens	Type of lens	Characteristics
L1	SV Org. 1.5 (SPH 0.00)	Organic resins: CR-39; Single vision; 1.5 index; Without treatment
L2	Min STK 170 MC (SPH −9.00)	Mineral (glass); 1.7 index; Multicoated
L3	VIEW 1.56 HC (SPH + 5.00)	Organic resins; Hard Coat; 1.56 index
L4	Superfin Lc-cinzento céfil	Superfin Organic resins: CR-39; Photochromic in lens matrix; Céfil (anti-refletive)
L5	Min STK 150 MC (SPH + 4.00)	Mineral (glass); 1.5 index; Multicoated
L6	Min STK 160 MC (SPH + 5.00)	Mineral (glass); 1.6 index; Multicoated
L7	STK 160 TRB HMC (SPH + 4.00)	Organic resins; 1.6 index; Hard Multicoated; Anti-reflective; Photochromic (brown) in film
L8	Min STK 150 FOT MC (SPH + 4.00)	Mineral (glass); 1.5 index; Photochromic in lens matrix; Multicoated
L9	STK 174 CC (SPH −9.00)	Organic resins; 1.74 index; Anti-reflective
L10	SV Org. 1.5 AR (SPH + 5.00)	Organic resins; Single Vision; 1.5 index; Anti-reflective
L11	FSH Photobrown HMC (SPH + 4.00)	Organic resins; Finished single version; Photochromic; Hard Multicoat
L12	Indorganic 167AS Natural (SPH + 5.00)	Organic resins; Natural Anti-reflective
L13	Indorganic 16 AS – Céfir (SPH + 5.00)	Organic resins; Photochromic

A subset of the lenses incorporated anti-refractive coatings, hard coatings, and photochromic compounds. This grinding process involves transforming the original manufacturer-supplied lenses into customised spectacle lenses by shaping according to a specific geometry predefined by the optician. The grinding process uses water-cooled grinding wheels to trim the edges of the lenses to achieve the desired configuration. The residual lens fractions and wastewater resulting from this process were collected from an on-site retention tank of the machine (Visionix), for subsequent characterisation, as shown in Supplementary Fig. 12.

### Fourier transform infrared spectroscopy

The vibrational analysis of the mixture of 187 lenses was carried out using Fourier transform infrared spectroscopy (FTIR) by reflectance with the non-destructive sampling technique of attenuated total reflectance (ATR). Infrared spectra (4000–550 cm^−1^) of the sample, at room temperature, were recorded using a Perkin Elmer Frontier spectrometer (FT-NIR/MIR), equipped with an FR-DTGS detector and a KBr beam splitter. The spectra were recorded with a resolution of 4.0 cm^−1^ for a total of 128 accumulations (scans). A Perkin Elmer sampling accessory was used, a Universal Attenuated Total Reflectance (UATR) module with a diamond/ZnSe crystal, and a constant force of 110 N was applied to all the recordings. The sample was analysed without any treatment.

### Raman spectroscopy

The Raman spectra were recorded using a Horiba LabRam Evolution confocal microscope with a 532-nm laser, a 600 g/mm grating, and a ×100 objective, resulting in a laser spot diameter of ~ 0.5 μm.

To obtain the lens matrix spectrum, Raman measurements were performed on the middle of the cross-section face of the lens setting the confocal hole size to 200 mm to improve the signal-to-noise ratio. To obtain the spectra of the multi-coatings, however, the laser was focused through a ×100 objective to a 0.5 mm spot at the very edge of the cross-section and the confocal hole was set to 30 mm to reduce the contribution of the Raman signal from the out-of-focus areas.

### Scanning electron microscopy

A TESCAN VEGA 3 SBH—Easy Probe SEM (TESCAN GROUP, a.s., Brno – Kohoutovice, Czech Republic) with a tungsten-heated cathode was used to perform the microstructural analysis of the solid waste samples. The SEM images of the lens material after cutting were acquired at a working voltage of 5 kV using the secondary electron detector. Before analysis, the samples were coated with a 10 nm-thick gold/palladium layer using a Quorum SC7620–Mini Sputter Coater/Glow Discharge System (Quorum Technologies, Laughton, UK).

### Particle size distribution

The particle size distribution of ophthalmic solid waste was measured using a Mastersizer 2000 model (Malvern Instruments Ltd., Malvern, UK), with the Fraunhofer approximation. This equipment is suitable for the analysis, since its measurement range is 0.2 to 2000 µm. During analysis, the temperature was maintained at 25 °C, using water as a dispersant under agitation at 1500 rpm. An average of six replicates was determined for each sample. The presence of very small and agglomerate particles has been shown to increase the effective viscosity of the suspension, thereby distorting the laser diffraction reading. The effect of particle aggregation or dispersion is influenced by the balance between Van der Waals and electrostatic forces. The smaller the diameter of the nanoparticles, the greater the surface area, resulting in very high surface energy and making the particles unstable. Consequently, these particles tend to agglomerate to reduce the energy levels present. Therefore, ultrasound was used for disaggregating the particles. This approach facilitates the measurement of primary particles, thereby ensuring accuracy in the analysis. The duration of ultrasound treatment was 30 s, and the measurement was taken immediately afterwards.

The technique analyses the diffraction pattern generated by the interaction of laser light with the particles in the sample to determine particle size and distribution. The surface-weighted mean diameter, D[3,2], represents the average particle size based on each particle’s contribution to the total sample surface area, while the volume-weighted mean diameter, D[4,3], indicates the average size based on each particle’s contribution to the total sample volume. The D[4,3] and D[3,2] were calculated according to Eqs. (1) and (2), respectively, where *n*_i_ is the number of particles with a diameter *d*_i_ (“Understanding and Interpreting Particle Size Distribution Calculations”).

1 $$D\left[\mathrm{4,3}\right]=\frac{{\sum }_{1}{n}_{i}{d}_{i}^{4}}{{\sum }_{i}{n}_{i}{d}_{i}^{3}}$$

2 $$D[\mathrm{3,2}]=\frac{{\sum }_{i}{n}_{i}{d}_{i}^{3}}{{\sum }_{i}{n}_{i}{d}_{i}^{2}}$$

Additionally, d(0.1), d(0.5), and d(0.9) were calculated, which correspond to the 10th, 50th, and 90th percentiles of the particle’ cumulative size distribution. The relative span factors were determined using Eq. 3, to express the distribution width of the droplet size distribution:

3 $$\mathrm{Span}= \frac{d\left(0.9\right)-d(0.1)}{d(0.5)}$$

### Atomic absorption spectroscopy

Determination of the amounts of copper (Cu), cadmium (Cd), chromium (Cr), and lead (Pb) in the ophthalmic waste was performed using Perkin-Elmer PinAAcle 500 atomic absorption equipment (PerkinElmer, Inc., Shelton CT, USA). Perkin-Elmer Intensitron Hollow Cathode lamps were used at the following wavelengths: 324.75, 228.80, 238.32, and 357.87 nm, respectively. The equipment was calibrated using five standards of different concentrations (BDH Spectrosol grade, supplied by VWR International, Radnor PA, USA) for each element. Six replicates were performed for each element. The ophthalmic solid waste was subjected to chemical acid digestion using 6 M HCl at 150 °C.

### Energy dispersive X-ray analysis (EDS)

X-ray microanalysis of ophthalmic solid waste was conducted using the Bruker QUANTAX system, which incorporates the Bruker Nano XFlash® detector (Bruker Corporation, Billerica, Massachusetts, USA). This detector has an energy-dispersive X-ray detector, with an energy resolution of 133 eV (Mn Ka) at 100 kcps. It has an effective area of 10 mm^2^ and is cooled by a Peltier element. Elements ranging from B (Z = 5) to Am (Z = 95) can be identified and quantified. The software module uses a standardless PB-ZAF method for quantification. The system was installed in a TESCAN VEGA 3 SBH—Easy Probe SEM (TESCAN GROUP, a.s., Brno–Kohoutovice, Czech Republic). ESPRIT 1.9 Software was used for data analysis. The EDS analysis was performed on an uncoated sample.

### Differential scanning calorimetry (DSC)

The thermal properties of the different types of lenses were characterised using DSC, employing a DSC 214 Polyma system (NETZSCH-Gerätebau GmbH, Selb, Germany). The lenses were heated from 20 to 600 °C at a rate of 10 °C/min. All tests were carried out on samples weighing 5–7 mg, which were placed in aluminium crucibles; the empty crucible was used as a reference. The nitrogen flow rate was 40 mL/min.

The mixture of 187 lenses was thermally analysed using a TA Instruments, Discovery Series DSC25, which was calibrated at a heating rate of 5 °C/min using indium and tin standards. The temperature range was −20 to 250 °C with a heating rate of 10 °C/min. The test was performed on a sample of 5.1020 mg placed in an aluminium crucible, with an empty crucible used as a reference. The nitrogen flow rate was 100 mL/min.

### Thermogravimetric analysis

Thermal gravimetric analysis (TGA) was performed using a TA Instruments TGA Q500. The analysis employed a temperature range of 20 to 600 °C, with a heating rate of 10 °C/min. The test was performed on a sample weighing 5.3440 mg.

### Gas chromatography-mass spectrometry

A mixture of water and solid waste was obtained from the final ophthalmic waste resulting from lens cutting, and the different phases were separated by decantation. Liquid–liquid and solid–liquid extractions using dichloromethane were then conducted on the liquid and solid phases, respectively. The organic phases of both samples were concentrated using vacuum drying. The samples (0.3 µl) were injected (slipt flow 15 mL/min) into an Agilent 7890 A chromatographer (Agilent Technologies, Inc., Santa Clara, CA, USA). The analytes were separated using an HP-5MS column and identified using an MS Agilent 5975c detector. The carrier gas (helium) was maintained at a linear rate of 36.3 cm/s. The chromatographer’s heating method was as follows: 40 °C for 5 min, then heating to 220 °C at a rate of 10 °C/min and remaining at 220 °C for a further 5 min. The injector and interface temperatures were 250 and 240 °C, respectively.

The substances were identified using the W9N08.L library, and the results represent the closest matches.

### Nuclear magnetic resonance

1D ^1^H and 2D ^1^H-^1^H NMR DOSY (Diffusion-ordered spectroscopy) experiments were carried out on a 9.4 T Bruker AVANCE III 400 spectrometer (Bruker Corporation, Billerica MA, USA) equipped with a Bruker DiffBB probe, specifically a 5 mm high-power diffusion BBI probe. 1D ^1^H NMR spectra were acquired with zg30 pulse sequences with 32 k complex points, 32 scans, a spectral width of 8417.51 Hz, and a 1 s relaxation delay, at 25 °C. Pseudo-2D ^1^H-^1^H DOSY-NMR experiments were acquired using the diffusion bipolar pulse air-stimulated echo and LED (LEDBPGP) method with 32 k complex points and 16 scans per increment at a spectral width of 6009.62 Hz and 25 °C. Data were obtained with a diffusion delay of 400 ms, a small delta of 4 ms, and linear gradient pulse amplitudes ranging from 2 to 95% in 16 steps. An eddy current delay of 5 ms was also used. Temperature calibration was performed using standard samples. Bruker Topspin 3.5 was used to acquire and process all data.

### Statistical analysis

Statistical analysis was performed using Microsoft Excel (Microsoft Corp., USA). The relationship between refractive index and material waste was assessed through comparative mean analysis. Results are expressed as mean values ± SEM (n = 187). To assess significant differences in the percentage of mass loss among the 12 experimental groups, a single-factor analysis of variance (ANOVA) was performed. In order to compare the different categories of refractive indices, the lenses were grouped into three distinct classes: low index (1.50), medium index (1.56–1.60), and high index (1.67–1.74). Paired comparisons between these categories were performed using Student’s *t*-test for two samples assuming unequal variances (Welch’s test), since sample sizes and variances differed between groups. For all statistical tests, a confidence level of 95% was adopted, with results considered statistically significant when the probability value is less than 0.05.

## Results and discussion

### Estimated mass loss of ophthalmic lenses

**Fig. 2:**
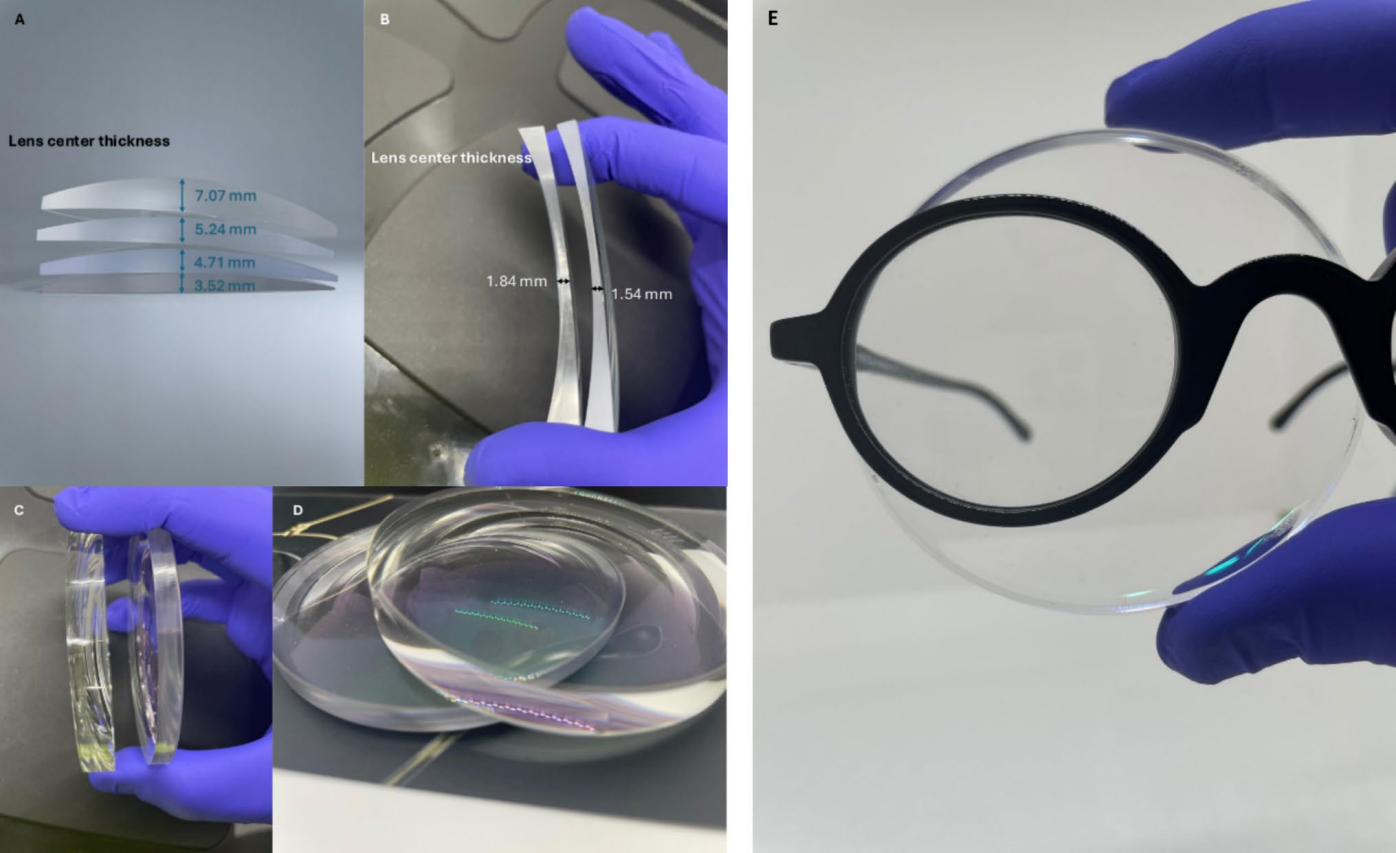
Examples of lens forms comparisons (categories of lens form: plus lenses and minus lenses). Four different types of lenses for the same plus lenses: + 5.00 D prescription with refraction indexes 1.74 (3.52 mm), 1.67 (4.71 mm), 1.60 (5.24 mm) and 1.50 (7.07 mm). **A** Two different types of lenses for the same minus dioptric: – 5.00 D prescription with refraction indexes 1.60 (1.84 mm) and 1.67 (1.54 mm). **B** Two different types of lenses for the same minus dioptric: – 5.00 D prescription with refraction indexes 1.50 D and 1.74 D (**C** and **D**); A comparative analysis of lens and eyeglass frame materials illustrating the proportion of lens waste generated per completed eyeglass (**E**)

This study involved cutting 187 lenses exhibiting different characteristics. These variations included the shape, spectacles size, and thickness of the lenses, as illustrated in Fig. 2. The material loss generated during lens grinding was investigated by considering these lens characteristics, including comparisons based on dioptric power and refractive index.

The comparison of plus lenses (+ 5.00 D) with different refractive indices (1.74, 1.67, 1.60, and 1.50) reveals a direct correlation between the lens’s centre thickness (3.52, 4.71, 5.24, 7.07 mm) and its refractive index. A lower refractive index (1.50) corresponds to a significantly thicker lens (7.07 mm) compared to a high-index lens (1.74), which is considerably thinner (3.52). Similarly, considering minus lenses (−5.00 D), a lower refractive index (1.60) corresponds to a higher lens centre thickness (1.84 mm) compared to a higher refractive index (1.67), which is thinner (1.54 mm). A thicker initial lens (plus and minus) requires more extensive grinding to achieve the final eyeglass frame form (Fig. 2E), which in turn generates a greater amount of waste. This reflects how the choice of lens material, considering its refractive index, is a determining factor in the volume of material discarded during the manufacturing process.

**Fig. 3:**
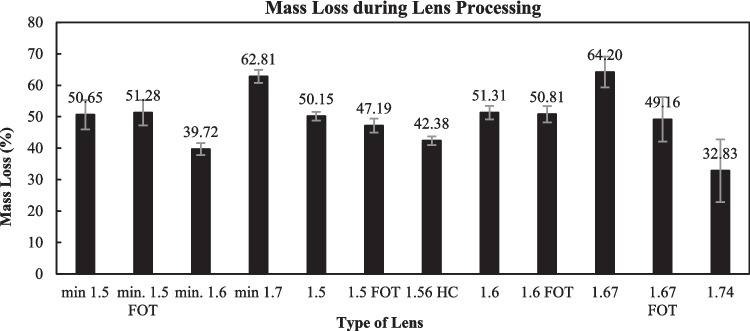
Average mass loss of spectacle lenses during grinding. A total of 187 lenses of 12 types were investigated. min (mineral, Crown glass); FOT (*photochromatic)* HC (*hard coated*). The values 1.5, 1.6, 1.7, 1.56, 1.67, and 1.74 are the refractive indexes

The average percentage of mass loss generated from the production of eyeglass lenses in the ophthalmic industry is represented in Fig. 3. The total average is 49%, indicating that approximately 50% of the initial lens material is discarded during the production of the final product.

According to the latest 2026 reports, the global market for spectacle lenses (comprising both glass and organic materials) reached between 900 million and 2.8 billion units in 2024 (“Ophthalmic Lens Market Report: Trends and Forecasts 2025–2033,” “Revenue of eyewear in the United States from 2018 to 2029, by product type,” “World—Spectacle Lenses Of Glass Or Other Materials—Market Analysis, Forecast, Size, Trends and Insights”). The same reports value the 2024 market size between USD 19.76 billion and USD 89.98 billion and the global average annual acquisition of spectacle lenses was approximately 0.34 to 0.45 units per capita. This data varies considerably depending on the specific market report methodology and particularly definitions and variables used. Therefore, accounting for these discrepancies and opting for a conservative estimate based on historical data, it can be inferred that with an average annual acquisition of 0.09 spectacle lenses per capita and a global population of 8 billion, the total annual lens production is approximately 720 million units.

Supplementary Fig. 1 compares the estimated total mass of lenses produced in 2023 and the corresponding waste generated during cutting. The waste mass per lens was determined by the difference between the initial (γ_IW_) and the final lens weight (γ_FW_). To estimate the total annual environmental burden, a conservative estimate approach was used. The average waste mass per lens was calculated by summing the average mass loss of each lens type and dividing by the total number of lens types. This average waste mass was then multiplied by the total annual lens production to estimate the annual waste. Considering an approximate 50% material loss, an estimated 5,770 tonnes are directly discharged into wastewater networks. This annual waste scale, in order of kilotonnes, underscores the substantial environmental challenge associated with releasing ophthalmic materials (including nano and microplastics) into wastewater systems.

The statistical analysis of the experimental data suggests that mass loss during the manufacturing process is not uniform and is significantly influenced by the intrinsic characteristics of the lenses, such as refractive index.

The results, presented in Supplementary Table 2, revealed a highly significant difference between the 12 subgroups (188,93F = 2.908; *p* = 0.0015). This suggests that the intrinsic characteristics of each lens subgroup such as the refractive index and material have influence on the volume of waste generated. ANOVA test demonstrates that the differences between materials (64.20% in index 1.67 vs. 32.83% in index 1.74) are sufficiently robust to be statistically validated.

The calculated F value (2.908) is greater than the critical F value (1.843). This means that the variability between groups is almost three times greater than the internal variability within groups, rejecting the null hypothesis of equality between means.

The value of *p* = 0.00151 is significantly lower than 0.05 suggesting the results are considered highly significant. There is a probability greater than 95% that the differences in mass loss are due to the technical characteristics of the lenses. After ANOVA validation, a more targeted analysis was performed using Student’s *t*-test for unequal variances. The purpose of the test was to assess whether the technical characteristics of the lenses, specifically their refractive indices (low, medium, and high indices), correlated with the volume of waste generated during grinding process. While a secondary ANOVA grouped by three macro categories (low, medium, and high indices) remained significant (*p* = 0.0178) (Supplementary Table 4), more targeted pairwise analyses using Student’s *t*-test for unequal variances provided a more in-depth analysis.

The results of the pairwise comparisons (Supplementary Table 3) indicate that, when grouped into these macro categories (low, medium, and high indices), the differences are no longer statistically significant (*p* > 0.05 in all comparisons). The comparison between low and high indices was the closest to significance suggesting a trend that could not be confirmed with two-tailed statistical rigour. The comparison between low and high indices lenses showed a higher mean mass loss for the high-index group (58.34% vs. 49.71%). However, this difference did not reach statistical significance at the 95% confidence level (*p* = 0.069, two-tailed *t*-test). Despite the lack of formal significance, a marginal trend was observed (one-tailed *p* = 0.035).

These results offer significant insights for market analysis and can drive research and technological advancements. Spectacle lens consumption is driven by various regional realities and consumer preferences (including vision impairments by region, socioeconomic status, professional requirements, and regional trends), all of which dictate lens type selection. However, the statistical analysis demonstrates that although there are variations in the volume of material removed by lens type, the generation of waste is inherently systemic. The production of this waste is not a result of consumer behaviour or choice; rather, it is a structural and systemic consequence, a technological constraint or limitation, inherent to the current optical subtractive processes.

While the data in this study were collected from a single optical store, the findings remain highly representative of the industry due to the high degree of standardisation in both lens materials and processing equipment. The global ophthalmic market relies on a limited number of polymer matrices (allyl diglycol carbonate, polycarbonates, and high-index polythiourethanes), and the subtractive grinding process is technologically uniform across different optical stores and countries. Variations between stores are expected primarily in the relative frequency of specific lens type, driven by regional fashion trends for frame sizes or consumer purchasing power, and in the chemical compositions of coatings.

### Characterisation of ophthalmic lenses by Raman spectroscopy

**Fig. 4:**
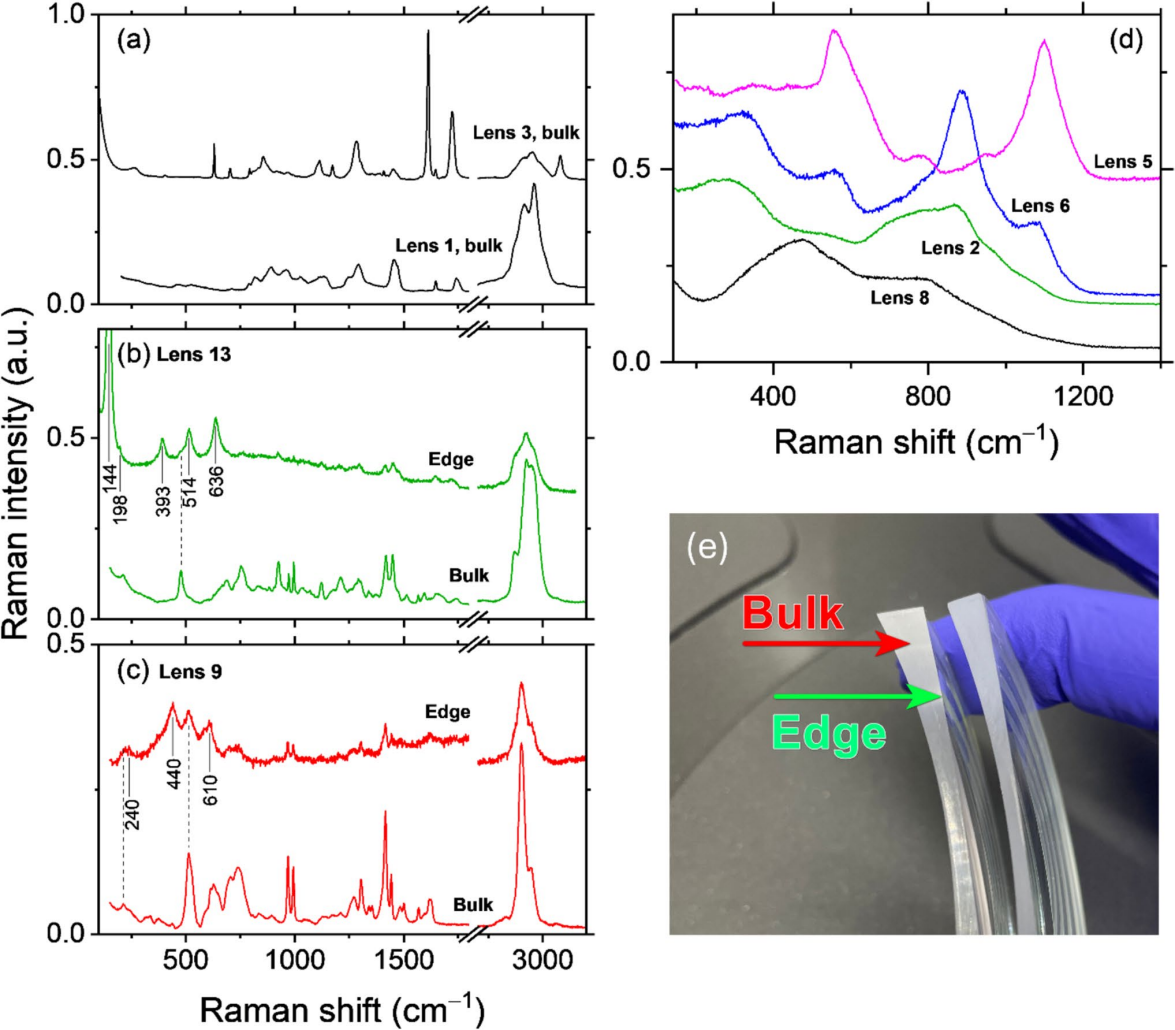
Raman spectra of organic (panels **a**, **b**, **c** 4 lenses total) and mineral lenses (**d** 4 lenses). For organic lenses, both bulk (polymeric matrix) and edge (multi-coating at the edge) spectra are measured from the polished cross-section face as shown in panel **e**. For mineral lenses (panel d), only bulk spectra are shown. The characteristic bands of anatase (panel **b**) and rutile (panel **c**) forms of TiO_2_ are marked by vertical solid lines with wavenumber labels. The dotted lines are guides for the eye. The spectra are vertically offset for clarity. The x-axis break in (**a**–**c**) separates the fingerprint region from the high-wavenumber region

Raman spectroscopy was employed to identify the chemical composition of lens materials, which is essential for understanding the environmental fate, degradation pathways, and potential toxicity of waste discharged during grinding (Bertoluzza et al. 1986). Accurate compositional analysis informs appropriate waste management strategies, as different polymer types and coating materials exhibit distinct environmental behaviours (Kawecki & Nowack 2019). The lenses comprised both mineral and organic polymeric matrices with different multicoatings. Although 13 lenses (12 types) were analysed, Fig. 4 shows representative spectra (four organic and four mineral) to illustrate the main spectral features. Raman spectroscopy readily distinguishes between polymeric and mineral matrices, revealing sharp bands across the entire fingerprint region for organic matrices (Fig. 4, panels *a-c*) compared to substantially broader and lower-energy Raman bands (typically below 1200 cm^–1^) associated with the amorphous nature of mineral components (Fig. 4, panel *d*). Thus, based on their general Raman profiles, out of 13 lenses studied, nine of them were organic, and four were mineral. Identification of the specific lens material was attempted by comparing the measured Raman spectra with published reference spectra. Only CR-39 (allyl diglycol carbonate polymer; lens 1 in Fig. 4a) and an optical polyester (lens 3, Fig. 4a) showed clear agreement with literature spectra (Bäumer, 2010; Shekhawat et al., 2011; Was-Gubala & Starczak 2015). The remaining polymeric lenses could not be assigned with confidence because the available published references did not yield an unambiguous match, including for common candidates such as polycarbonate, poly(methyl methacrylate) (PMMA), polypropylene, cyclic olefin copolymers, and polysulfone (Bäumer, 2010; Bertoldo Menezes et al. 2021; de Menezes et al. 2021; S. N. Lee et al. 2000). This limitation is expected, since ophthalmic lenses typically contain co-monomers and various additives/fillers that modify optical, mechanical, and chemical properties (G. Suri et al. 2009). Consequently, Raman-based material identification is inherently challenging for these lenses, because formulation additives/coatings and co-monomer mixtures can alter or mask diagnostic bands relative to published reference spectra, preventing a reliable match.

For mineral matrices, one lens spectrum was consistent with an aluminosilicate glass of approximate composition 75SiO_2_·25Na_2_O·2Al_2_O_3_ (lens 5, Fig. 4d) (Yadav & Singh 2015).

Concerning the multicoating layers, only TiO_2_ was identified, which was found to be present in both anatase and rutile polymorphic forms. TiO_2_ oxide was detected at the edge of the cross-section faces in four out of 13 lenses studied (Fig. 4e). Figure 4 presents the Raman spectra of lenses 9 and 13 from both bulk and edge areas. The spectra from the edges of the lenses exhibit characteristic anatase (at 144, 198, 393, 514, and 636 cm^–1^) and rutile (240, 440, and 610 cm^–1^) features for lens 13 and 9, respectively, in addition to the Raman bands of the bulk lens material (see Fig. 4b and c). The detected Raman signals of TiO_2_ likely originate from antireflection coatings, where this oxide is commonly employed alongside other materials such as ZrO_2_, MgF_2_, SiO_2_, among others (Cao et al. 2022; Eigenmann et al. 1998; Schottner et al. 2003). Despite the typical layer thickness of a multilayer AR coating being only around one-quarter of the visible wavelengths (Cao et al. 2022; De, Jana, Medda, & De, 2013), TiO_2_ is detectable due to its strong Raman scattering. The contributions of other multilayer coatings constituents to the overall Raman spectrum appear negligible, likely attributable to their significantly weaker Raman cross-sections and minimal thicknesses. The identification of TiO_2_ in both anatase and rutile crystalline forms in the lens coatings is environmentally significant. Titanium dioxide nanoparticles are recognised as emerging environmental pollutants with documented ecotoxicological effects, including oxidative stress induction in aquatic organisms and disruption of microbial communities (Dedman et al. 2021; Rashid et al. 2021; Rathore et al. 2023; Sibiya et al. 2025; Simonin et al. 2016). The distinct spectral signatures observed between the bulk lens material and the edge regions demonstrate the compositional heterogeneity of lens‑grinding wastewater matrix, comprising both organic polymers and inorganic metal oxide nanoparticles, which presents challenges for waste treatment and necessitates comprehensive multi-component characterization approaches.

### Characterisation of the lens-grinding waste

Solid waste generated from the grinding of 187 lenses was collected from an optical store, and subsequently characterised for particle size distribution, homogeneity, and composition. This collected material represents typical waste produced by optical stores, which, as previously mentioned, is currently disposed of through the wastewater system due to a lack of pertinent legislation and established reuse protocols.

#### Structural and morphological characterisation of lens waste

**Fig. 5:**
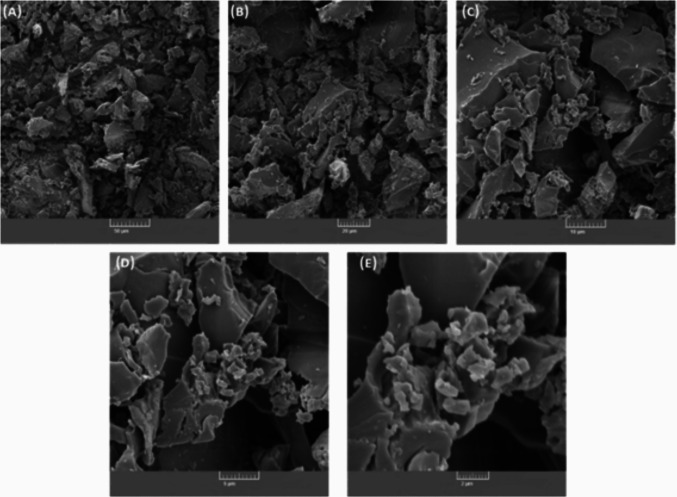
Scanning electron microscopy (SEM) micrographs of the solid waste samples, with increasing amplification: **A** × 500; **B** × 1000; **C** × 2500; **D** × 5000; **E** × 10,000

Figure 5 illustrates the heterogeneous nature of particle dimensions, observed across varying magnifications using Scanning Electron Microscopy (SEM). This heterogeneity can be attributed to the lens processing method, where the combination of lens cutting and water jet action produces a wide range of particle sizes, resulting in a highly heterogeneous surface topography. Figure 5E demonstrates the presence of sub-2 µm particles, which pose a particular risk to the environment and public health. These fine particles can penetrate many water filtration systems, potentially contaminating water reservoirs and initiating a bioaccumulation process that may ultimately affect food chains and human health.

To further characterise the particle size distribution, the Fraunhofer method was employed. The lens-grinding waste was analysed both before and after ultrasound treatment. Ultrasound treatment was employed to disaggregate and disperse any potential agglomerates present in the sample, thereby improving measurement consistency and ensuring the measurement of individual particles. Supplementary Table 5 presents the particle size distribution of residue, including d[0.1], d[0.5], d[0.9], span, D[4,3], and D[3,2].

The initial graph in Supplementary Fig. 2, illustrating the particle size distribution of lens-grinding waste before ultrasound treatment, shows a broad size range from 0.01 to 3000 µm. The asymmetrical curves indicate a heterogeneous distribution with significant variation in nano- and microplastic size. The presence of multiple peaks suggests the existence of distinct particle size populations. The objective of the ultrasound treatment was to assess whether there was a reduction in particle size and an improvement in dispersion homogeneity after treatment (second graph of Supplementary Fig. 2). A comparative analysis of the two curves, corroborated by the data presented in Supplementary Table 5, reveals a decrease in average particle size, as reflected by the D[3,2] and D[4,3] values, suggesting particle disaggregation. An increase in distribution homogeneity is also observed, as indicated by the reduced span value, which signifies a more uniform particle size distribution. The decrease in d[0.9] confirms the reduction of larger microplastics, likely due to fragmentation or the dispersion of agglomerates. This reduction in particle size enhances sample homogeneity and, consequently, elevates the surface area. Smaller size exhibits a higher surface-to-volume ratio, increasing the interface available for environmental interactions. Furthermore, this high surface area accelerates dissolution and adsorption processes. However, the resulting thermodynamic instability often triggers particle agglomeration as a mechanism to minimise total surface energy.

This, in turn, can enhance properties such as reactivity, solubility, and adsorption capacity, potentially advantageous for various applications. Furthermore, the reduction in particle size may optimise processes such as filtration, sedimentation, and drying.

Chemical digestion was employed to decompose ophthalmic waste to quantify heavy metals. The concentrations of copper, cadmium, chromium, and lead in the ophthalmic lens waste were determined, and data are presented in Supplementary Table 6. Chromium toxicity is valence state-dependent, with Cr (VI) being significantly more toxic than Cr (III). Consequently, exposure to Cr(VI) poses risks of lung, nasal, and sinus cancers, respiratory diseases, and skin irritation (WHO 2019a). In the case of chromium, exposure to this heavy metal can induce pulmonary diseases, kidney damage, ocular irritation, among other adverse effects. Lead exposure is associated with development and neurobehavioural effects on fetuses, infants, and children. In adults, lead is linked to elevated blood pressure (WHO 2019b). Considering the estimated 5,770 tonnes of lens material discarded into the environment, we can extrapolate that approximately 36,351 kg of Cr, 66,932 kg of Pb and 577 kg of Cd are released. While the quantities may appear insignificant on a global environmental scale, our primary concern lies with the occupational exposure of individuals working in optical stores. These workers are daily exposed to particulate matter during lens cutting, a process where inhalation of these particles is not entirely preventable.

Energy dispersive X-ray spectroscopy (EDS) was employed to determine the elemental composition of the solid waste generated from processing a sample of 187 lenses. The results revealed that solid waste is primarily composed of carbon, oxygen, and sulfur (see Supplementary Table 7). Furthermore, other chemical elements, including nitrogen and silicon, were detected. These elements are consistent with the expected composition of common ophthalmic lens polymers, such as polycarbonates, polystyrenes, polythiourethanes, and silica-containing components.

#### Thermal properties and decomposition behaviour analysis

The solid waste was also analysed using differential scanning calorimetry (DSC). This technique provides information based on glass transition temperatures and polymer crystallinity, which can affect their properties and potential environmental impact. As depicted in Supplementary Fig. 3, an endothermic peak centred around 100 °C indicates a melting process, suggesting a solid-to-liquid phase transition. Beyond this temperature, the observed baseline decrease may be attributed to the material’s heat capacity and other thermal properties.

In addition to DSC, thermogravimetric analysis (TGA) provides complementary information. TGA offers insights into the thermal stability of microplastics and their potential degradation behaviour in the environment. Furthermore, TGA can reveal the presence of additives and filters in microplastics, as these components may exhibit distinct thermal properties compared to the polymeric matrix or base polymer. Different polymers also display varying degradation patterns based on decomposition characteristics and mass loss profiles. The thermogravimetric analysis (TGA) curve for the ophthalmic waste exhibits a slight initial mass loss at lower temperatures, likely due to the evaporation of moisture or other volatile compounds in the sample (Supplementary Fig. 4). A more substantial mass loss, occurring at approximately 200 °C, indicates thermal decomposition or degradation of the material. Beyond this decomposition event, the TGA curve continues to show a gradual mass decrease, possibly attributable to oxidation and decomposition at elevated temperatures.

#### Chemical analysis of lens waste

The ophthalmic waste was further characterised using FTIR-ATR spectroscopy. Despite the sample’s heterogeneous composition, which includes various lens types, presenting challenges in definitively assigning all functional groups, several characteristic peaks were observed. In Supplementary Fig. 5, a weak peak at 3338 cm^−1^ corresponds to N–H stretching vibrations, potentially associated with polythiourethanes present in some ophthalmic lenses. The absorption between 2700 and 3000 cm^−1^ is indicative of aliphatic C–H stretching. Peaks corresponding to C = O and C = C stretching vibrations are observed at approximately 1700 and 1600 cm^−1^, respectively. A strong peak at approximately 1250 cm^−1^ suggests the presence of C-O stretching vibrations. Finally, = C–H vibrations were identified around 750 cm^−1^, indicating the presence of aromatic compounds in the sample.

Prior to analysis, a preliminary treatment was conducted to facilitate the examination of the solid waste generated during lens edging. The edging process results in a mixture of water and solid residues, which were separated by decantation. To identify the compounds present in both the liquid and solid phases, liquid–liquid and solid–liquid extractions were performed using dichloromethane (3 × 20 mL) at room temperature. The organic phases from the extractions were then subjected to vacuum drying. GC–MS spectra of both samples enabled the identification of various compounds (see Supplementary Fig. 6 and 7). The spectral fingerprints of the identified compounds, along with their names (derived from spectral libraries) and molecular weights, are presented in Supplementary Tables 8 and 9. Although both samples originated from the same lens grinding waste mixture, GC–MS analysis revealed distinct compositional profiles. In general, the compounds identified across both analyses exhibited a broad range of molecular weights, spanning from 100 to 1000 g/mol. Notably, the compounds identified in the solid waste differed from those found in the wastewater, suggesting variations in compound solubility between aqueous and organic phases. The solid–liquid extraction sample exhibited a higher proportion of aromatic structures, generally exceeding 90%. In contrast, the liquid phase sample showed the presence of fluorine-containing compounds, but at lower relative percentages.

To validate the GC–MS results, extracts from both the solid waste and wastewater underwent further analysis using nuclear magnetic resonance spectroscopy, including diffusion-ordered spectroscopy (DOSY) and ^19^F NMR. The molecular weights of the identified structures ranged from 100 to 1000 gmol^−1^ for both samples (Supplementary Tables 10 and 11), consistent with the GC–MS data, except compounds D, E, I, and J, which exhibited higher molecular weights. This discrepancy could be attributed to molecular aggregation in solution. Aromatic compounds, prevalent in solid waste, are known to undergo π-π interactions in solution due to overlapping aromatic ring orbitals, which can promote intermolecular attraction. The precise compositional characterisation of the samples is challenging due to the extensive range of observed molecular weights. Supporting the GC–MS findings, the spectra (Supplementary Fig. 8) show a less pronounced presence of aromatic signals in the 7–8 ppm region for the wastewater sample compared to the solid waste (Supplementary Fig. 10). Conversely, characteristic signals of aliphatic compounds are more prominent in the wastewater sample (Supplementary Fig. 8).

The GC–MS analysis suggested the presence of fluorine-containing compounds, particularly in the wastewater sample. To validate this, ^19^F NMR was conducted. As illustrated in Supplementary Fig. 9 and 11, both samples exhibited signals indicative of fluorinated structures. The wastewater sample (Supplementary Fig. 9) showed two distinct peaks, consistent with the GC–MS identification of heptadecyl heptafluorobutyrate and heptadecyl pentafluoropropionate. On the other hand, the ^19^F NMR of solid waste showed a single peak, confirming the presence of 9-decen-1-ol, trifluoroacetate, which aligns with the GC–MS results (retention time = 24.056 min).

The diffusion coefficients and corresponding molecular weight ranges determined are consistent with the expected compounds present in the solutions (compounds A and H), particularly the fluorinated compounds confirmed by the ^19^F NMR spectra.

In summary, the results obtained from the various analyses conducted during the study provided a comprehensive picture of the lens composition. In the RAMAN analysis of the various lenses, the identification of the constituent elements of each layer of the lenses was hindered by the reduced thickness of the lenses. However, the analysis did reveal the presence of TiO_2_ in the form of rutile and anatase, a constituent that is recognised as a component of lens coatings and which poses a threat to the environment. Subsequent analyses of the resulting material from these lens cuts yielded further insights, including the size of the particles, which were found to be sub-2 µM. These particles exhibited a propensity to aggregate due to their size-related instability. Furthermore, the presence of heavy metals, phthalates and bisphenol A was identified by GC–MS analysis. Another characterisation technique that yielded interesting data was NMR, which enabled the identification of compounds containing fluorine. These compounds present a significant health risk due to their ease of introduction into the food chain and the environment.

The significant environmental impact of eyewear lens production, together with the absence of specific regulations in this sector, has raised concerns among professionals in the field, particularly opticians, who are advocating for the implementation of effective mitigation strategies. Furthermore, addressing water consumption during lens edging is critical, necessitating a comprehensive assessment of current water resource availability and the development of sustainable practices to minimise the environmental footprint of lens manufacturing.

The increasing accumulation, environmental persistence, and widespread distribution of deleterious lens-grinding waste demand urgent mitigation strategies to minimise adverse environmental impacts. This can be achieved through improved cleaning and wastewater treatment, alongside exploring alternative materials that prioritise waste recovery and integration into a circular economy to create valuable products (Wilhelmi 2022). The circular economy, focusing on sustainability and resource efficiency, extends product lifecycles and reduces waste (Dziuba et al. 2021). One waste reduction approach involves minimising primary lens mould production and standardising spectacle frames for precise lens manufacturing, thus decreasing shaping needs, water consumption, and waste. However, this is challenging without regulatory intervention due to diverse spectacles styles. A more practical alternative is developing value-added products from this waste, such as materials for 3D printing or injection moulding. For instance, our previous research demonstrated the fabrication of cementitious mortars and polymer-based composites from ophthalmic waste collected using a closed-circuit edging machine (Encarnação et al. 2024). Similarly, another study explored the potential of ophthalmic glass lens waste as an alternative soluble silica source for alkaline-activation reactions. Specifically, glass powder was employed as a substitute for commercial sodium silicate in the production of fly ash-based alkaline cement (Najafi et al. 2023). Excessive water consumption could be mitigated through the implementation of equipment with closed-loop water circuits. However, most of the commercially available equipment lacks this functionality. A straightforward modification to enable water collection and reuse would drastically reduce water consumption, mirroring established practices in water treatment facilities.

## Results in perspective: legal and regulatory considerations for ophthalmic lens waste

As previously stated, market projections indicate an increase in demand and production of spectacles and lenses, owing to the rising prevalence of individuals with refractive errors, advanced age, and excessive use of digital equipment. This phenomenon is also anticipated to generate an increase in waste, thereby exacerbating an existing problem that has been previously identified and the solution to which is currently being sought. Drawing upon the comprehensive research conducted in this study, it was possible to detect the presence of chemical compounds in the composition of lens-cutting waste, which pose a significant threat to living beings and the environment. These include TiO_2_, fluorine compounds, bisphenol A, heavy metals such as cadmium, lead, and chromium, polythiourethanes, among others, which are particularly hazardous and have the potential to compromise ecosystems.

While a specific regulation for this specific waste is absent from the regulatory landscape, the EU has a set of regulations, directives, and action plans to tackle pollution and accelerate its reduction. European Green Deal, Circular Economy Action Plan, Zero Pollution Action Plan, The European Strategy for Plastics in Circular Economy, “Restore our Ocean and Waters by 2030,” “Directive concerning urban wastewater treatment” are some of these documents that address water pollution.

For instance, the Directive (EU) 2024/3019, concerning urban wastewater treatment, recognises that, as regards to non-domestic wastewater, including industrial wastewater commercial establishments, or hospitals and other medical facilities “there is a poor understanding and poor knowledge of such pollution, which can lead to a deterioration in the functioning of the treatment process and contribute to the pollution of the receiving waters”(“Directive (EU) 2024/3019 of the European Parliament and of the Council of 27 November 2024 concerning urban wastewater treatment,”). This same directive revises that “Member States should regularly monitor and report on such non-domestic pollution that enters urban wastewater treatment plants and is discharged into water bodies”. Additionally, the document also reports that “where non-domestic pollution is identified in the incoming waters, Member States should take appropriate measures to reduce pollution at source, by enhancing the monitoring of pollutants in collecting systems so that the pollution sources can be identified.” It is recommended that Member States undertake a risk identification and assessment process in relation to urban wastewater management. This process should be followed by the implementation of preventive measures to limit the risk of microplastics, whether intentionally or unintentionally released, reaching urban wastewater and sludge (“Directive (EU) 2024/3019 of the European Parliament and of the Council of 27 November 2024 concerning urban wastewater treatment,”).

Also, the EU Action Plan: “Towards Zero Pollution for Air, Water and Soil” (12 May 2021) sets key 2030 targets which include a reduction by 50% plastic litter at sea and by 30% microplastics released into the environment (“Communication from the Commission to the European Parliament, the Council, the European Economic and Social Committee and the Committee of the Regions Pathway to a Healthy Planet for All EU Action Plan: “Towards Zero Pollution for Air, Water and Soil””). A further finding was the presence of particles, some of which were plastic and measured on the nanometre scale. These particles could potentially be breathed in. Opticians are exposed to particles during lens cutting, even though the environment is closed, representing a potential risk to the respiratory system and lead to illness. The absence of integration of waste from the optical industry into the classifications of the directives or handling guidelines drawn up by the EU represents a lack in current legislation. In the absence of regulations, this waste is often disposed of in the environment.

A crucial first step in tackling pollution is identifying and characterising the residues discharged in urban wastewater. With robust data and evidence on this specific waste, information can be provided to policymakers to help them develop adequate regulations. Another crucial aspect of tackling the problem is to find alternative solutions for this residue. The absence of adequate solutions for the collection of this waste, coupled with the lack of legislation mandating the monitoring and control of such materials (which, as has been demonstrated in this study, contain toxic compounds and are in microscale), compels opticians to dispose of surplus materials through the wastewater system. It is incumbent upon each Member State to recognise this underestimated source of pollution, regulate the industry, and promote a shift towards more sustainable materials and processes.

## Concluding remarks and recommendations

This study provides a comprehensive characterisation of the wastewater generated during the ophthalmic lens-grinding process, using a representative sample of 187 lenses of 12 different types. The findings reveal a highly heterogeneous residue characterised by a significant fraction of sub-2 µm particles and potentially hazardous components, including heavy metals and complex fluorinated substances.

The repercussions of this industry have not been thoroughly explored, and there are no studies that have identified the actual problem. The optical sector is frequently overlooked in discussions regarding environmental microplastics and chemical pollution. Currently, a substantial portion of this waste is discharged directly into municipal wastewater systems, posing underestimated risks to both aquatic ecosystems and public health. Our findings, based on conservative estimates, reveal that 5,770 tonnes were directly discharged into wastewater networks in 2023.

To visualise the environmental impact and illustrate the scale of this unknown waste stream: based on material density (Encarnação et al. 2024) and ISO 20’ standard marine container specifications, the estimated annual tonnage would fill 206 marine containers. If we extrapolate this data over the year, the optical sector would be filling and discarding a marine container every 42 h. Furthermore, if we consider the data from the latest reports, these figures would be 3.5 times higher. This represents a massive, concentrated flow of nano and microplastics and other pollutants, and it exposes the urgent need for alternative waste management systems at the retail level to prevent this massive, fragmented discharge into the oceans.

Despite the growing awareness and demand for a solution among professionals in the sector, the absence of specific legislation and accessible collection infrastructure remains a critical barrier to sustainable waste management.

To address these challenges, future research must prioritise the development of efficient on-site treatment technologies capable of mitigating the risks associated with micro- and nano-sized contaminants. Furthermore, scientific inquiry should explore viable pathways for the reuse and recycling of lens-grinding residues, promoting a circular economy within the optics industry. Finally, the implementation of targeted regulations and responsible disposal protocols is imperative to ensure environmental integrity and safeguard the occupational health of those engaged in the optical industry.

## Supplementary Information

Below is the link to the electronic supplementary material.

ESM 1 (DOCX 1.50 MB)

## Data Availability

The authors declare that the data supporting the findings of this work are available within the manuscript and its Supplementary Information files. Should any data files be needed in another format, they are available from the corresponding author upon reasonable request.

## Declarations

### Clinical trial number

This is not applicable.
