# Supplementary material for: Physical and chemical characterisation of ophthalmic lens-grinding wastewater: uncovering environmental implications
Source: Environ Sci Pollut Res Int. 2026 Apr 1;33(14):6493–508. doi: 10.1007/s11356-026-37708-w (PMC13124795; doi:10.1007/s11356-026-37708-w)
Supplement: Supplementary file 1 — (DOCX 1.50 MB) [file 11356_2026_37708_MOESM1_ESM.docx]

**Supplementary Material**

**Supplementary Table 1.** Descriptive statistics of material waste by lens type. Data represent the percentage of mass loss across 12 experimental groups (n=187). Values are presented as mean (Average %), median, and standard error of the mean (SEM). FOT and HC denote specific material or processing variants.

| Type of Lenses | n | Average(%) | Median (%) | SEM |
| --- | --- | --- | --- | --- |
| min 1.5 | 7 | 50,649 | 53,565 | 4,651 |
| min. 1.5 FOT | 11 | 51,282 | 52,948 | 4,014 |
| min. 1.6 | 4 | 39,716 | 38,523 | 1,911 |
| min 1.7 | 2 | 62,806 | 62,806 | 2,072 |
| 1.5 | 59 | 50,153 | 48,135 | 1,361 |
| 1.5 FOT | 20 | 47,191 | 47,850 | 2,205 |
| 1.56 HC | 4 | 42,376 | 41,284 | 1,378 |
| 1.6 | 53 | 51,305 | 53,472 | 2,152 |
| 1.6 FOT | 2 | 50,806 | 50,806 | 2,591 |
| 1.67 | 19 | 64,201 | 72,509 | 4,884 |
| 1.67 FOT | 2 | 49,163 | 49,163 | 7,105 |
| 1.74 | 4 | 32,832 | 29,820 | 9,949 |

### **Supplementary Table 2.** Results of the single-factor analysis of variance (ANOVA) for percentage mass loss for the 12 lens groups analysed.

| *Source of Variation* | *SS* | *df* | *MS* | *F* | *P-value* | *F crit* |
| --- | --- | --- | --- | --- | --- | --- |
| Between Groups | 6062,2998 | 11 | 551,118164 | 2,90794388 | 0,00151844 | 1,84370828 |
| Within Groups | 33166,2792 | 175 | 189,521595 |  |  |  |
|  |  |  |  |  |  |  |
| Total | 39228,579 | 186 |  |  |  |  |

*The table shows the overall comparison between the 12 lens groups analysed (n=187). The F value (2.908) greater than the F critical value (1.843), together with a p-value of 0.0015 indicates that there are statistically significant differences between the mean mass loss values of the groups at a 95% confidence level.*

*SS: sum of squares; df: degrees of freedom; MS: mean square.*

**Supplementary Table 3.** Pairwise comparisons between refractive index categories using Student's t-test.

| *Paired Comparisons* | *t Stat* | *P(T≤t) one-tail* | *P(T≤t) two-tail* | *t Critical*  *two-tail* | *Result* |
| --- | --- | --- | --- | --- | --- |
| Low Index vs. Medium Index | -0,285 | 0,388 | 0,776 | 1,983 | No Significance |
| Medium Index vs. High Index | -1,662 | 0,053 | 0,106 | 2,032 | No Significance |
| Low Index vs. High Index | -1,888 | 0,035 | 0,069 | 2,048 | No Significance |

*The lenses samples were grouped into three macro categories of refractive indices: Low Index (1.50), Medium Index (1.56-1.60) and High Index (1.67-1.74). Student's t-test was applied to two categories assuming unequal variances (Welch's test). The results show that, despite the trends observed, the differences between the index categories did not reach statistical significance (p>0.05), suggesting that waste is transversal across all ranges of materials.*

### **Supplementary Table 4.** Results of the single-factor analysis of variance (ANOVA) for percentage mass loss grouped into three macro categories of refractive indices.

| ANOVA |  |  |  |  |  |  |
| --- | --- | --- | --- | --- | --- | --- |
| *Source of Variation* | *SS* | *df* | *MS* | *F* | *P-value* | *F crit* |
| Between Groups | 1679,86554 | 2 | 839,93277 | 4,11592344 | 0,01783646 | 3,04503999 |
| Within Groups | 37548,7135 | 184 | 204,069095 |  |  |  |
|  |  |  |  |  |  |  |
| Total | 39228,579 | 186 |  |  |  |  |

**Supplementary Fig.1.** Comparison between the total lenses produced and the resulting ophthalmic waste in 2023.

**Supplementary Fig.2.** Particle size distribution of lens-grinding waste before and after undergoing ultrasound treatment obtained using laser diffraction. Each curve represents a different measurement for the specified condition.

**Supplementary Table 5.** Particle size distribution parameters (span and characteristic diameters) of lens-grinding waste, determined by laser diffraction using the Fraunhofer approximation. Values represent the average of multiple measurements.

|  | D[3,2]µm | D[4,3]µm | d[0.9]µm | d[0.5]µm | d[0.1]µm | span |
| --- | --- | --- | --- | --- | --- | --- |
| Lens-grinding waste | 9.14 (±0.48) | 70.52 (±7.94) | 153.03 (±20.65) | 33.38 (±3.16) | 4.02 (±0.33) | 4.45  (±0.18) |
| Lens-grinding waste after submitting to ultrasounds | 7.119 (±0.008) | 53.260 (±0.897) | 118.286 (±0.953) | 23.530 (±0.121) | 2.730 (±0.008) | 4.911 (±0.018) |

**Supplementary Table 6.** Quantification of heavy metals in ophthalmic waste by atomic absorption spectroscopy.

| Sample | mg Cu/g | mg Cd/g | mg Cr/g | mg Pb/g |
| --- | --- | --- | --- | --- |
| Ophthalmic waste | 0.0069 | ≤0.0001 | 0.0063 | 0.0116 |

**Supplementary Table 7.** Elemental analysis by EDS of ophthalmic residues of edging lenses.

| Element | Normalized Content (wt. %) | Error (wt. %) |
| --- | --- | --- |
| Carbon | 59.17 | 16.27 |
| Nitrogen | 9.13 | 4.53 |
| Oxygen | 11.66 | 4.26 |
| Silicon | 0.36 | 0.12 |
| Sulfur | 19.68 | 1.49 |


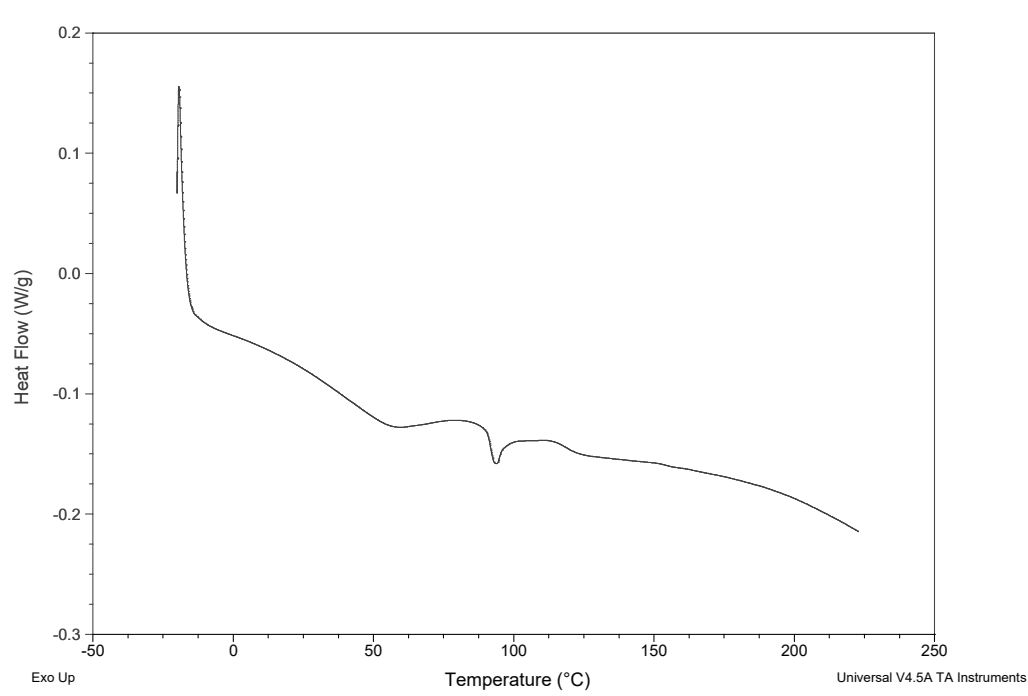


**Supplementary Fig. 3.** Differential scanning calorimetry characterisation of lens-grinding waste.


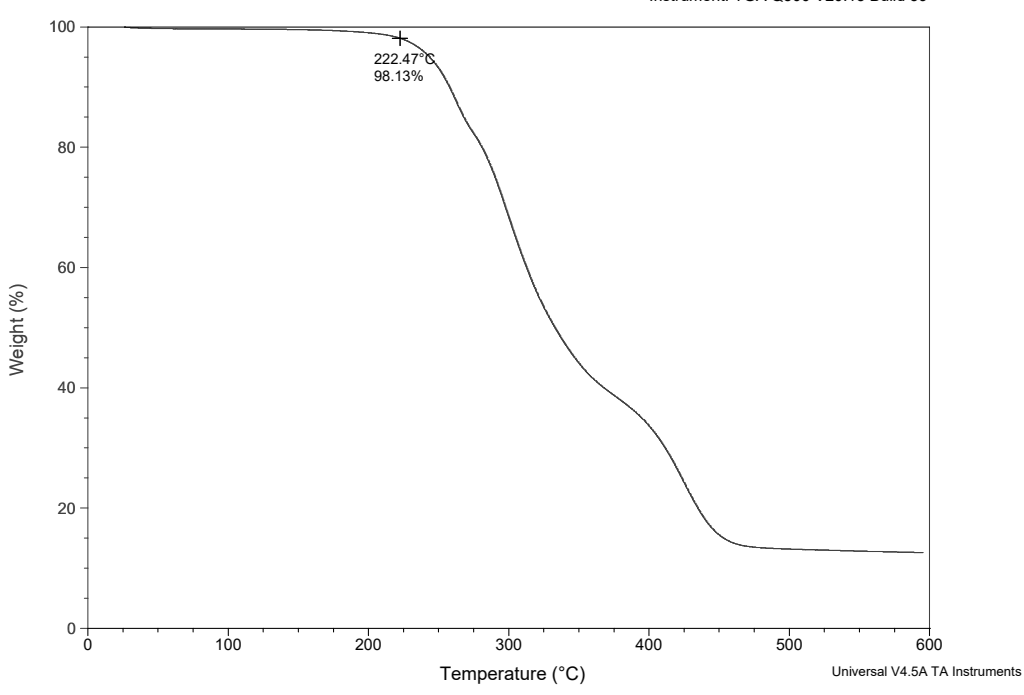


**Supplementary Fig. 4.** TGA characterisation of lens-grinding waste.

**Supplementary Fig. 5.** FTIR characterisation of lens waste.

**
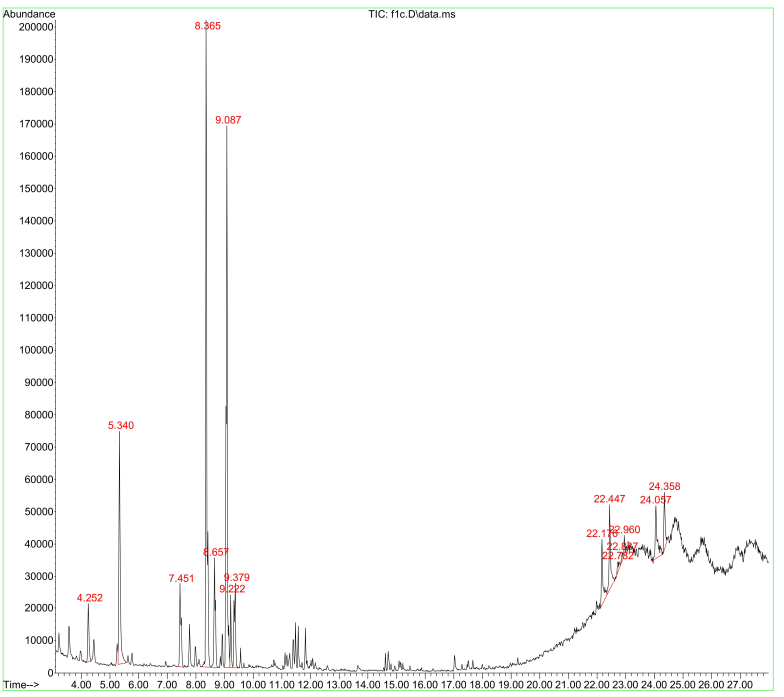
**

**Supplementary Fig. 6.** GC-MS chromatogram of the ophthalmic lens wastewater obtained in the final stage of 187 eyeglass lens grinding.

**Supplementary Table 8.** Compounds identified by GC-MS in ophthalmic wastewater.

| **Retention time** | **Name of Compound** | **MW in g/mol** | **Formula** | **% Similarity** |
| --- | --- | --- | --- | --- |
| 4.252 | Formyl pyruvyl urea | 158.11 | C_5_H_6_N_2_O_4_ | 64 |
| 5.340 | 4-methyl-2-pentanol | 102.17 | C_6_H_14_O | 59 |
| 7.471 | 2,4-dimethyl-3-hexene | 112.21 | C_8_H_16_ | 43 |
| 8.365 | Trans-2-pentenal | 84.12 | C_5_H_8_O | 64 |
| 8.657 | Diisoamyl ether | 158.28 | C_10_H_22_O | 64 |
| 9.087 | 1-Methyl-2-propylcyclohexane | 140.27 | C_10_H_20_ | 80 |
| 9.222 | 3,3,4-trimethyl-hexane | 128.25 | C_9_H_20_ | 53 |
| 9.379 | 3-ethyl-3-heptanol | 144.25 | C_9_H_20_O | 38 |
| 22.176 | Heptadecyl pentafluoropropionate | 402.49 | C_20_H_35_F_5_O_2_ | 76 |
| 22.447 | 2-(methoxymethyl)-1,1,3,3-tetramethyl-1,3-disilacyclohexane | 216.47 | C_10_H_23_OSi_2_ | 72 |
| 22.782 | Sulfurous acid, butyl octadecyl ester | 390.7 | C_22_H_46_O_3_S | 83 |
| 22.887 | Nonahexacontanoic acid | 999.83 | C_69_H_138_O_2_ | 76 |
| 22.960 | Nonahexacontanoic acid | 999.83 | C_69_H_138_O_2_ | 64 |
| 24.057 | HAHNFETT  Phytol acetate | ---  338.57 | ---  C_69_H_138_O_2_ | 81  72 |
| 24.358 | Heptadecyl heptafluorobutyrate | 452.5 | C_21_H_35_F_7_O_2_ | 76 |

**
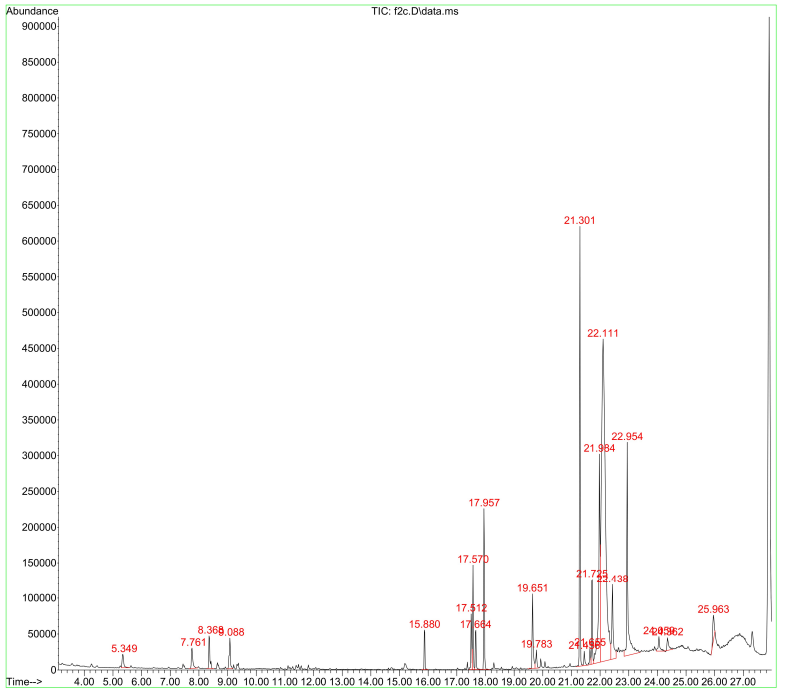
**

**Supplementary Fig. 7.** GS-MS chromatogram represents the analysis of the solid ophthalmic waste in the final stage of 187 eyeglass lens grinding.

**Supplementary Table 9.** Compounds identified by GC-MS in solid ophthalmic waste.

| **Retention time** | **Name of Compound** | **MW in g/mol** | **Formula** | **% Similarity** |
| --- | --- | --- | --- | --- |
| 5.349 | 2-Hexanol | 102.17 | C_6_H_14_O | 59 |
| 7.761 | Styrene | 104.15 | C_8_H_8_ | 95 |
| 8.368 | 3,4-Dihydropyran | 84.12 | C_5_H_8_O | 59 |
| 9.088 | Sulfurous acid, cyclohexylmethyl pentadecyl ester | 388.60 | C_22_H_44_O_3_S | 64 |
| 15.880 | Octanoic acid-tert bytyl ester | 200.32 | C12H24O2 | 37 |
| 17.512 | meso-2,3-Diisobutyl-2,3-dimethylsuccinic acid dinitrile | --- | --- | 72 |
| 17.570 | 2,6-di-t-butyl-4-methylene-2,5-cyclohexadiene-1-one | 218.33 | C_15_H_22_O | 99 |
| 17.664 | meso-2,3-Diisobutyl-2,3-dimethylsuccinic acid dinitrile | 219.37 | C_13_H_24_N_2_ | 72 |
| 17.957 | 2,6-bis(1,1-dimethylethyl)-4-methyl-phenol | 220.35 | C_16_H_24_O | 97 |
| 19.651 | 2H-Imidazole-2-thione, 1,3-dihydro-1-methyl- | 114.17 | C_4_H_6_N_2_S | 50 |
| 19.783 | 6,6-Dimethylcycloocta-2,4-dienone | 150.22 | C_10_H_14_O | 43 |
| 21.301 | 2,4-diphenyl-4-methyl-1-pentene | 236.35 | C_17_H_18_ | 96 |
| 21.436 | Cis-para-methane | 140.27 | C_10_H_20_ | 30 |
| 21.655 | Diallyl terephthalate | 260.29 | C_14_H_14_O_4_ | 74 |
| 21.725 | 2,4-Diphenyl-4-methyl-2(E)-pentene | 236.35 | C_18_H_20_ | 97 |
| 21.984 | N-(Anilinocarbothioyl)valine | --- | --- | 46 |
| 22.111 | 2-(2H-benzotriazol-2-yl)-4-(1,1,3,3-tetramethylbutyl)phenol (ultraviolet absorvent UV-329) | 323.43 | C_20_H_25_N_3_O | 86 |
| 22.438 | 1,1,2,2-tetramethyl-1,2-digermacyclopentane | 247.50 | C_7_H_18_Ge_2_ | 56 |
| 22.954 | Dibutyl phthalate | 278.34 | [C_16_H_22_O_4_](https://pubchem.ncbi.nlm.nih.gov/#query=C16H22O4) | 93 |
| 24.056 | 11,13-Dimethyl-12-tetradecen-1-ol acetate  9-Decen-1-ol, trifluoroacetate | 282.46  252.27 | [C_18_H_34_O_2_](https://pubchem.ncbi.nlm.nih.gov/#query=C18H34O2)  C_12_H_19_F_3_O_2_ | 74  68 |
| 24.662 | 3-(2,5-Dimethyl-1H-pyrrole-3-yl)-1,3-dihydro-indol-2-one | --- | --- | 90 |
| 25.968 | Bisphenol A | 228.29 | C_15_H_16_O_2_ | 98 |


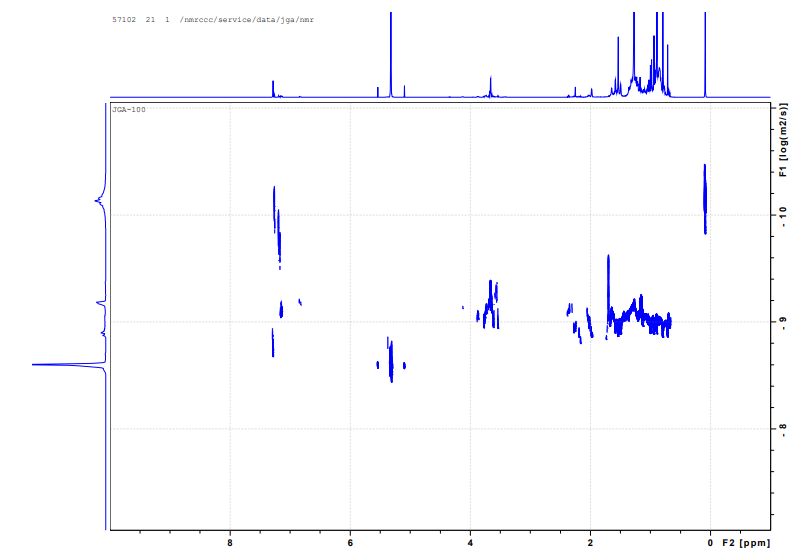


E

D

C

B

A

**Supplementary Fig. 8.** ^1^H DOSY NMR of ophthalmic wastewater obtained in the final stage of 187 eyeglass lens grinding.

**Supplementary Table 10.** The measurement of ophthalmic wastewater was done at 25ºC in deuterated chloroform. ^a 1^H DOSY NMR experiment. ^b^ Hydrodynamic radius calculated via the Stokes-Einstein equation D = k_B_T/6πƞr_H_ (k_B,_ Boltzmann constant; T, absolute temperature; ƞ, viscosity of CDCl_3_ at 298 K); * Molecular weights above 1000 g/mol suggest aggregation in solution.

| Compound | Diffusion coefficient (10-10 m2/s) a | Hydrodynamic radius (nm)b | Molecular weight (g/mol) |
| --- | --- | --- | --- |
| A | 8.8 | 1.58 × 10-9 | 173 |
| B | 9.1 | 7.94 × 10-10 | 413 |
| C | 9.17 | 6.76 × 10-10 | 944 |
| D | 9.7 | 1.99 × 10-10 | 18296* |
| E | 10.01 | 9.77 × 10-11 | 129031* |


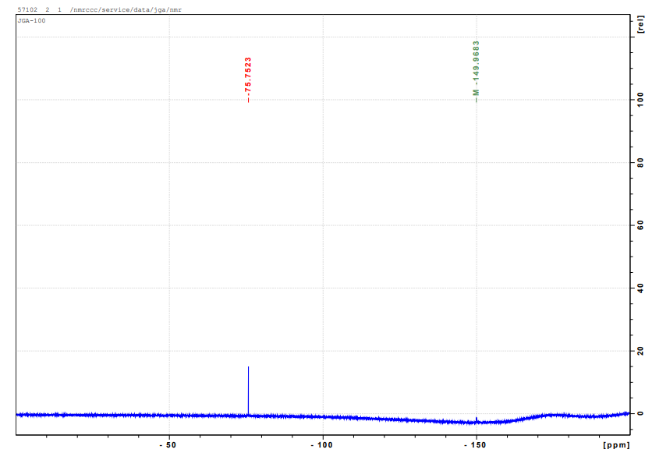


**Supplementary Fig. 9.** ^19^F NMR of ophthalmic wastewater obtained in the final stage of 187 eyeglass lens grinding.

**Supplementary Fig. 10.** ^1^H DOSY NMR of solid ophthalmic waste obtained in the final stage of 187 eyeglass lens grinding.

**Supplementary Table 11.** The measurement of solid ophthalmic waste was done at 25ºC in deuterated chloroform. ^a 1^H DOSY NMR experiment. ^b^ Hydrodynamic radius calculated via the Stokes-Einstein equation *D* = *k*_B_*T*/6*πƞr*_H_ (*k*_B,_ Boltzmann constant; *T*, absolute temperature; *ƞ*, viscosity of CDCl_3_ at 298 K); * Molecular weights above 1000 g/mol suggest aggregation in solution.

| Compound | Diffusion coefficient (10^-10^ m^2^/s) ^a^ | Hydrodynamic radius (nm) ^b^ | Molecular weight (g/mol) |
| --- | --- | --- | --- |
| F | 8.8 | 1.58 × 10^-9^ | 246 |
| G | 9.06 | 8.71 × 10^-10^ | 550 |
| H | 9.13 | 7.41 × 10^-10^ | 773 |
| I | 9.8 | 1.58 × 10^-10^ | 33991* |
| J | 9.9 | 1.26 × 10^-10^ | 64703* |


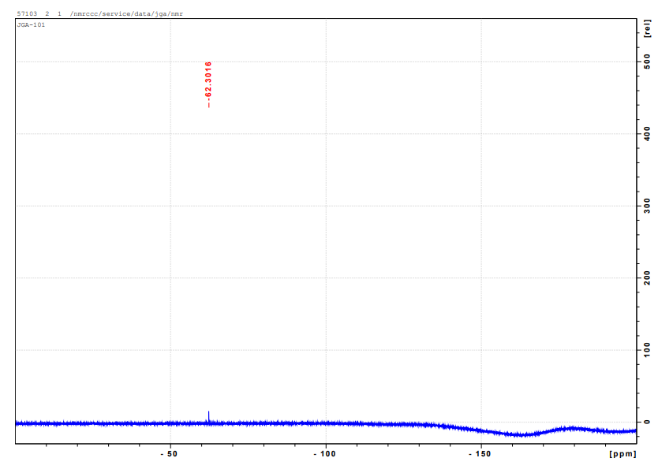


**Supplementary Fig. 11.** ^19^F NMR of solid ophthalmic waste obtained in the final stage of 187 eyeglass lens grinding.


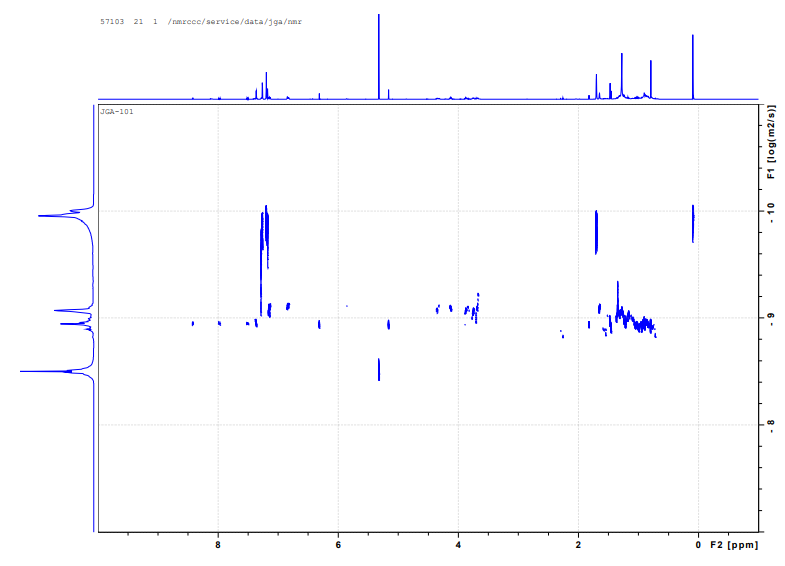


J

I

H

G

F


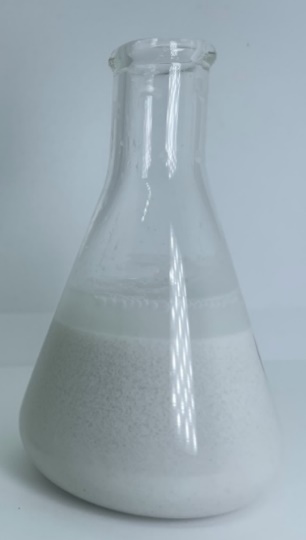


**A**


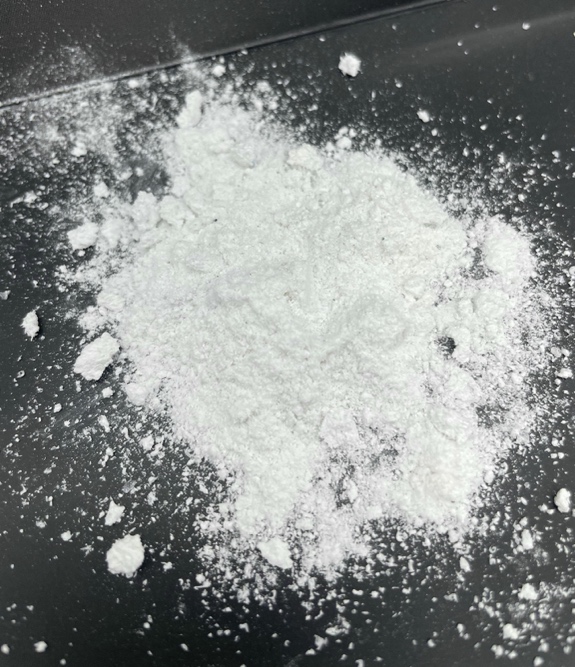


**B**

**Supplementary Fig. 12.** Optic industry wastewater supernatant obtained in the final stage of eyeglass lens grinding (**A**) and solids from ophthalmic spectacle lens wastewater (**B**).

**Supplementary Table 12.** Technical specifications of ISO 20' standard marine container and volumetric analogy.

| ISO 20' standard marine container | Parameter | Value | Source/Note |  |  |
| --- | --- | --- | --- | --- | --- |
| Container Dimensions | Internal Length | 5,900 mm | ISO 20' Standard |  |  |
|  | Internal Width | 2,352 mm | ISO 20' Standard |  |  |
|  | Internal Height | 2,395 mm | ISO 20' Standard |  |  |
| Container Capacity | Internal Volume | 33.2 m^3^ | Calculated |  |  |
|  | Max Payload | 28,130 kg | ISO 20' Standard |  |  |
| Waste Characteristics | Material Density | *1.3 t/m^3^* | <https://doi.org/10.3390/ma17010075> | |  |
| Annual Scale | Total Waste | *5,770,000 kg* | Calculated |  |  |
|  | Container Count | 206 Units | (Total Waste / Max Payload per Unit) | |  |
|  | Extended Scale | 721 Units | Based on 3.5x multiplier  (accordingly latest reports) | | |

* The number of containers was determined by dividing the total estimated annual waste by the structural weight limits of maritime transport (Max Payload) of a standard ISO 20' container (28,130 kg). This ensures the analogy accounts for the structural weight limits of maritime transport.
